# Fully Automated Plane Prescription in Cardiac MRI: A Prospective Cohort Study

**DOI:** 10.1002/jmri.70178

**Published:** 2025-11-30

**Authors:** Benjamin Böttcher, Felix G. Meinel, Karolin K. Deyerberg, Lena‐Maria Watzke, Mathias Manzke, Margarita Gorodezky, Gaspar Delso, Antonia Dalmer, Anne Nerger, Marc‐André Weber, Ann‐Christin Klemenz

**Affiliations:** ^1^ Institute of Diagnostic and Interventional Radiology, Pediatric Radiology and Neuroradiology Rostock University Medical Center Rostock Germany; ^2^ GE HealthCare Munich Germany; ^3^ GE HealthCare Barcelona Spain

**Keywords:** artificial intelligence, automated workflow, cardiac

## Abstract

**Background:**

Accurate plane positioning is important for high‐quality cardiac MRI images but requires specialized training, limiting accessibility.

**Purpose:**

To evaluate an automated plane positioning tool and compare it with manual planning.

**Study Type:**

Prospective.

**Population:**

Fifty‐seven healthy volunteers (28 males; median age 42 years) and 20 consecutive patients (15 males; median age 61 years) scheduled for clinical cardiac MRI.

**Field Strength/Sequence:**

Steady state free precession cine sequence at 1.5 T.

**Assessment:**

In volunteers, short‐axis (SAX), 2‐chamber (2CH), 3‐chamber (3CH), and 4‐chamber (4CH) cine images were acquired using both automated and manual prescription. Two blinded radiologists (5 and 6 years of clinical cardiac MRI experience) rated plane quality on a Likert scale (1 = *nondiagnostic* to 5 = *excellent*). Mean plane angle differences between manual and automated prescriptions were calculated. Left and right ventricular end‐systolic volume (ESV), end‐diastolic volume (EDV), stroke volume (SV), and ejection fraction (EF) were compared. In patients, the number of required manual corrections to automated prescriptions was recorded.

**Statistical Analysis:**

Wilcoxon matched‐pairs signed rank tests and Bland–Altman analyses, significance level at *p* ≤ 0.05.

**Results:**

Automated plane positioning was successful in all volunteers. Image plane quality did not differ significantly between automated (mean score 4.64) and manual prescription (4.62, *p* = 0.812). Mean angle differences were 6.7° ± 4.3° (SAX), 10.3° ± 5.8° (2CH), 8.9° ± 5.1° (3CH), and 8.0° ± 4.8° (4CH). Volumetric parameters showed no significant differences between both planning methods with mean biases being −0.5 mL, *p* = 0.305 (LVEDV), 0.5 mL, *p* = 0.683 (LVESV), −1.0 mL, *p* = 0.168 (LVSV) and 0.4%, and *p* = 0.215 (LVEF). In patients, 8.8% (7/80) of automatically prescribed planes required minor corrections; 91.2% (73/80) were accepted without adjustments.

**Data Conclusion:**

Automated plane positioning for cardiac MRI may provide high‐quality images and accurate volumetric assessment comparable to manual planning.

**Evidence Level:**

2.

**Technical Efficacy:**

Stage 2.

## Plain Language Summary

Accurate plane positioning is important for high‐quality cardiac MRI images and requires special training. This study evaluated an automated prescription method versus manual planning in 57 healthy volunteers and 20 patients. In healthy subjects, both methods provided good to excellent quality of planes without significant differences. Volumetric parameters from automatically planned images highly correlate with those from manual planes showing minimal bias without significant deviations. In patients, 91% of automatically prescribed image planes required no manual adjustments, and only minor changes were needed in the remaining 9%. Automated plane positioning may provide high‐quality images promising to simplify cardiac MRI acquisition.

## 1 Introduction

Cardiac MRI plays an important role in the assessment of a variety of diseases [1] and is implemented using imaging guidelines from international societies [2, 3, 4, 5]. To achieve and maintain diagnostic value, cardiac MRI relies on established standard imaging planes [6], which form the basis of recommended post‐processing steps [7] including the volumetric assessment of the left ventricle (LV) and right ventricle (RV). The interpretability and applicability of normal reference values of the heart chambers established by expert consensus [8], require the consistent utilization of these standard imaging planes. Therefore, plane prescription during a cardiac MRI examination is important for ensuring diagnostic image quality and comparability between follow‐up scans and different centers [9].

Conventional transverse, coronal, and sagittal image planes do not provide optimal visualization of cardiac structures. Consequently, the prescription of cardiac planes is a task that necessitates specialized training of technologists to ensure sufficient quality of scans [10]. Furthermore, the planning procedure requires time and is susceptible to errors and variability. These factors contribute to the limited availability of cardiac MRI for patients.

A semiautomated approach to simplify and accelerate plane prescription was developed in 2004 [11], but limited computational resources delayed further development. Initial fully automated plane positioning methods [12, 13, 14] have shown promising results but have required additional planning sequences, extending standard imaging protocols. Recent advances in the field of artificial intelligence (AI) have enabled the development of novel approaches to fully automated plane prescription without the need for manual preprocessing or additional localizer sequences while offering substantial time savings [15, 16, 17]. However, previous studies have involved retrospective investigations [15, 17] or small prospective cohorts in experimental settings [16]. The assessment of automated plane positioning approaches in a clinical setting using tools suitable for routine clinical use is currently lacking.

Thus, the aim of this study was to evaluate a fully automated AI‐based plane prescription approach with full integration into the MRI scanner in comparison to manual planning.

## 2 Material and Methods

### 2.1 Ethical Approval and Participants Selection

This prospective single‐center cohort study was approved by the responsible institutional review board. Written informed consent was obtained from each subject, before enrollment.

The study cohort included 57 healthy individuals recruited among employees and medical students (healthy cohort) and 20 consecutive patients receiving a clinically indicated cardiac MRI study (patient cohort). Inclusion criteria for both cohorts were: (1) ≥ 18 years of age and (2) provided written informed consent. Exclusion criteria were: (1) suspected pregnancy, (2) general contraindications for MRI, such as claustrophobia or non‐MRI‐suitable implants, and for the healthy cohort (3) any pre‐existing conditions that may directly or indirectly alter the cardiac structure or function (e.g., diseases of the cardiovascular system, chronic metabolic diseases, chronic respiratory diseases or rheumatic diseases).

The healthy cohort included equal numbers of males and females, and there were at least three males and three females in each of the following pre‐defined age groups: ≤ 34 years, 35–44 years, 45–54 years, and ≥ 55 years of age. Demographic data were collected in advance of the MRI study and the heart rate was recorded by the scanner during examination.

### 2.2 MRI Protocol

Each subject underwent cardiac MRI on a 1.5 T MRI scanner (Signa Artist, GE HealthCare, Chicago, United States) with ECG‐triggering. First, fully automated plane prescription using a prototype AI‐based plane positioning tool (AIRxHeart Prototype, GE Healthcare, Chicago, United States) was used to acquire accelerated fast imaging employing steady state acquisition cine‐sequences (Sonic DL, GE HealthCare, Chicago, United States) utilizing three heart beats per slice acquisition (3RR) and breath holds [18] in standard imaging planes: a short‐axis (SAX) image stack and single imaging planes in 2‐chamber (2CH), 3‐chamber (3CH) and 4‐chamber (4CH) views. The automatically prescribed image planes were always accepted and no manual correction was performed. The standard imaging planes were then acquired with manual planning following established imaging guidelines [6], using the same accelerated (3RR) cine‐sequence (Sonic DL, GE HealthCare, Chicago, United States) with breath holds. Manual planning was performed by four technicians with varying levels of experience: two with at least 20 cardiac MRI exams and two with at least 100 cardiac MRI exams performed. During manual planning, the technologists were not blinded to the automatically prescribed image planes planned in the first part of the protocol but were not allowed to review the images acquired in those planes. MRI‐protocol parameters were: field of view: 340 mm^2^; in‐plane resolution: 1.7 × 1.5 mm^2^; pixel matrix: 200 × 224; slice thickness: 8 mm; spacing: 10 mm; frames/cardiac cycle: 30; TR: 3.3 ms; TE: 1.2 ms; flip angle: 55°; and acceleration: 6. The number of breath holds required for the SAX image stack depended on the heart rate and averaged 3; for long axis views a single breath hold maneuver was applied.

### 2.3 Automated AI‐Based Plane Prescription

The prototype tool for automated plane prescription (AIRxHeart Prototype, GE HealthCare, Chicago, United States) is based on a set of deep learning U‐Net models trained to localize anatomical landmarks such as the left ventricular apex or mitral valve utilizing heat map optimization [15]. The 3D localizer sequence serves as the initial source sequence for the automated planning tool to prescribe a transverse multislice sequence. Next, the newly acquired transverse sequence is used as the source sequence to prescribe a pseudo‐2CH and pseudo‐4CH view. These image stacks are utilized afterwards as source sequences to prescribe the SAX view. All long‐axis views (2CH, 3CH, and 4CH) are then planned using the SAX as the primary input sequence. Once the algorithm has identified the landmarks within the input image stack, the new imaging plane is positioned in accordance with these landmarks, in alignment with the recommendations for established standard imaging planes [6]. The software is comprised of multiple stages, and information is transferred between these stages and from one plane to the next as part of the planning process. While single planes are utilized for the detection of a given landmark, landmarks are propagated through the planning process, and multiple planes are employed for plane prescription. Accordingly, the automated planning process is equivalent to that of a human operator. The prototype was trained on over 1000 datasets including geographically diverse data, pediatric data and additional image contrasts, such as 3D cine sequences and black blood sequences. Additionally, intensity augmentation was applied to the training data using variations of frequency filtering techniques to enhance the training process. This development was based on models developed by Blansit et al. [15]. At present, the tool is capable of automated planning of the transverse image stack, as well as pseudo‐2‐chamber, pseudo‐4‐chamber, short‐axis, 2‐chamber, 3‐chamber, and 4‐chamber views.

The prototype is integrated into the MRI scanner user interface, allowing for comprehensive implementation into the technician's workflow. Once an initial 3D localizer and a multislice transverse sequence have been acquired, the user may select the source images and the desired plane orientation (e.g., the pseudo‐4‐chamber image stack to plan the short‐axis image stack). The selection is then confirmed, and the plane prescription is performed fully automatically. To continue the acquisition, the user simply needs to start the acquisition process. The selection of the source and target views can be performed fully automatically if the protocol set up is configured for this purpose. The user is able to change the automated plane orientation at any time, which was not allowed in the current study.

### 2.4 Subjective Image Assessment and Plane Angulation Discrepancies

In the healthy cohort, the subjective image plane position quality was assessed by two radiologists (B.B. with 5 years and A.D. with 6 years of experience in clinical cardiac MRI). The quality rating was performed using an in‐house developed software tool. All image series were exported in DICOM format and presented by the software in random order blinded to the acquisition mode (automated prescription vs. manual planning). The radiologists were asked to rate the quality of image plane positioning on a 5‐point Likert scale with 1 indicating “*nondiagnostic*,” 2 “*poor*,” 3 “*sufficient*,” 4 “*good*,” and 5 “*excellent*” quality. The quality ratings were based on the anatomical structures displayed in each cardiac image plane. Structures that must be completely and clearly visible as well as structures that must not be displayed were pre‐defined for each standard cardiac image plane following commonly accepted criteria, such as full depiction of the left and right atria and ventricles and the exclusion of the LVOT in the 4CH view. Separately, image quality was rated by the following pre‐specified criteria: image contrast of myocardium to blood pool, image sharpness (absence of blurring), and absence of artifacts using the same 5‐point scale. The results of the subjective image quality assessment were analyzed for all image orientations combined and for each image plane separately.

A reacquisition analysis was performed to evaluate how many image planes would need to be reacquired in a clinical scenario due to insufficient or nondiagnostic image plane positioning quality. Therefore, the number of image planes that received a 1/5 (“*nondiagnostic*”) or 2/5 (“*poor*”) quality rating by at least one of the two independent readers was collected for each cardiac view separately and overall acquired views. Subsequently, percentages of cases with a need for a reacquisition were calculated.

The angulation differences between manual and automated planned image planes were determined in two steps. First, the image orientation of the DICOM arrays was extracted with respect to the physical coordinate system defined as the direction matrix. Second, the angle between the two images was calculated using the dot product between the two normalized plane vectors. The mean and standard deviation of the absolute value of the measured angles were calculated for each image plane separately over all data sets.

### 2.5 Volumetric Analysis

Volumetric assessment of the LV and RV was performed on SAX image stacks using established post‐processing software (cvi42, version 5.16, Circle Cardiovascular Imaging Inc., Calgary, Canada). Further, biplane volumetric assessment was conducted for the LV to incorporate long axis image planes into the functional analysis. Short‐axis volumetric assessment was performed for LV and RV, whereas the biplane volumetric assessment was only performed for LV using 2CH and 4CH imaging planes. Detection of systole and diastole, epi‐ and endocardial contours of the LV as well as endocardial contours of the RV were detected automatically by the software. LV papillary muscles were incorporated into the blood pool. Subsequently, all contours and the definition of systole and diastole were reviewed and revised by a cardiovascular radiologist (B.B.) with 5 years of experience in clinical cardiac MRI, following recommendations [7, 9]. This radiologist was not blinded to the planning mode. However, the expert was not permitted to review any contours or volumetric results of the alternative planning mode. The following LV and RV volumetric parameters were assessed: end‐diastolic volume (EDV), end‐systolic volume (ESV), stroke volume (SV), and ejection fraction (EF). Additionally, LV diastolic mass was measured.

### 2.6 Application of Automated AI‐Based Plane Prescription to a Clinical Patient Cohort

All scans were performed on the same 1.5 T MRI scanner (Signa Artist, GE HealthCare, Chicago, United States) and using the same protocol parameters as reported in the MRI protocol section. The performance of the automated plane prescription was documented in terms of total planning failures (defined as a failure to generate an imaging plane or an automatically generated image plane that necessitates a complete manual replanning), necessity for manual corrections and type of manual corrections when applicable.

### 2.7 Statistical Analysis

Statistical analysis was performed using GraphPad Prism (version 10.3.1, GraphPad Software, Boston, United States). Demographic data is summarized using median and range. Normality testing with the Shapiro–Wilk test showed that the majority of LV and RV volumetric parameters were not normally distributed. Therefore, nonparametric Wilcoxon matched pairs signed rank tests with a level of significance of *p* ≤ 0.05 were used to evaluate differences between volumetric values obtained from manual and fully automated planned images. A correlation analysis between manual and automated derived volumetric parameters was performed with the nonparametric Spearman correlation analysis. Bland–Altman analysis of LV biplane, as well as of LV and RV short‐axis volumetric results was performed to identify the bias and 95% limits of agreement (LoA) between manual and fully automated planning.

## 3 Results

### 3.1 Study Population

The healthy cohort consisted of 57 healthy volunteers (28 males, 29 females) with a median age of 42 years (range: 23–71 years), median weight of 76 kg (range: 50–100 kg) and median height of 1.75 m (range: 1.59–1.96 m). The median heart rate recorded by the MRI scanner was 68 bpm (range: 44–101 bpm). All participants were in sinus rhythm and no rhythm abnormalities were recorded during the scans.

**FIGURE 1:**
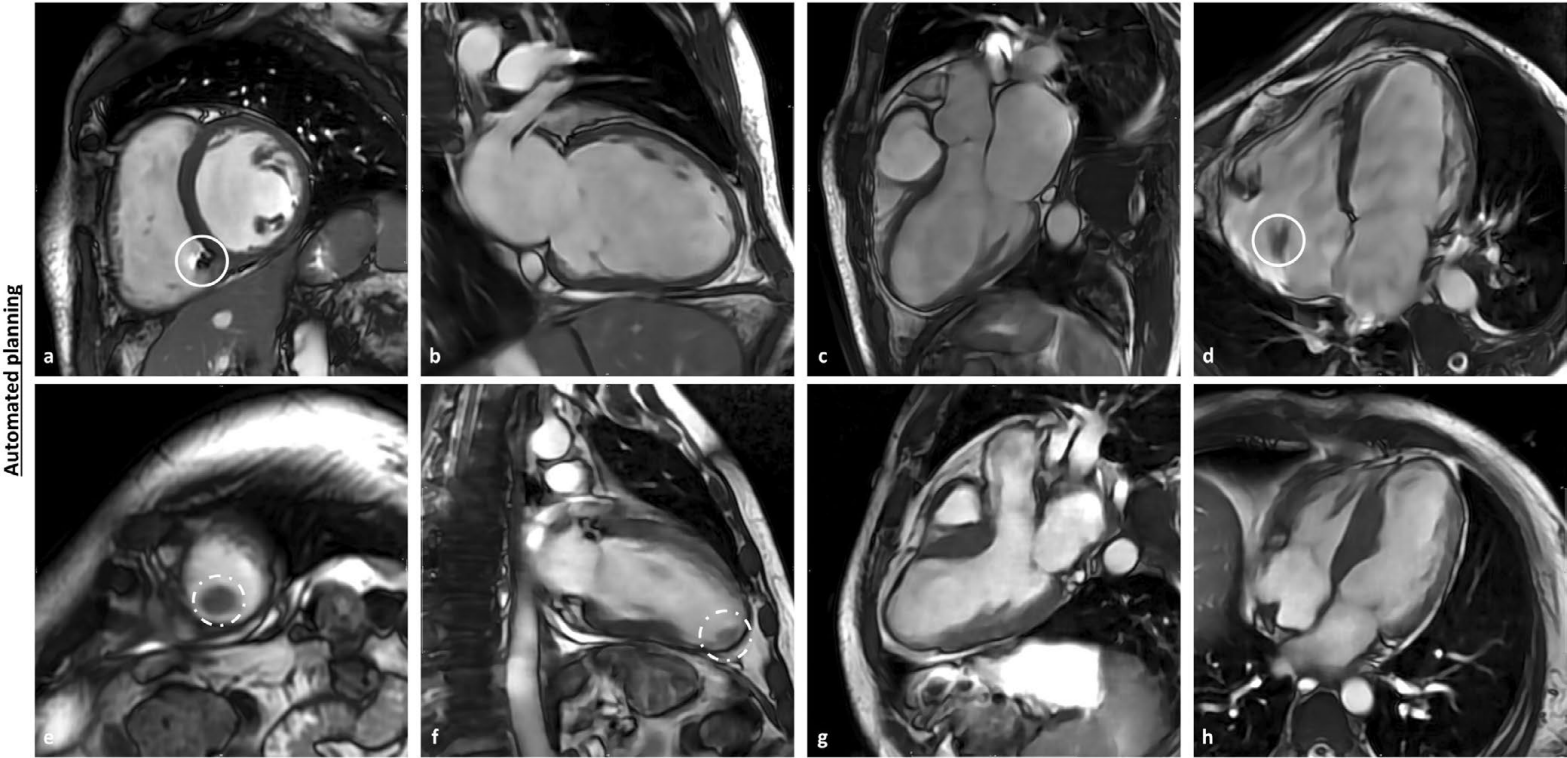
Example images acquired with fully automated plane prescription in patients with structural cardiac abnormalities. The upper row (a–d) displays SAX, 2CH, 3CH, and 4CH views of a 58‐year‐old male patient with dilated cardiomyopathy and a pacemaker device. The white ring marks the pacemaker wire, as well as local minor image artifacts. The bottom row (e–h) shows SAX, 2CH, 3CH, and 4CH images in a 48‐year‐old male patient with an apical left ventricular wall aneurysm. The white dashed ring marks an intraventricular thrombus adjacent to the aneurysmatic LV apex. Fully automated planning was successful for all planes in both cases without the need for manual adjustments. 2CH, 2‐chamber‐view; 3CH, 3‐chamber‐view; 4CH, 4‐chamber‐view; LV, left ventricle; SAX, short‐axis.

In the patient cohort, 20 patients (15 males, 5 females) with a median age of 63.5 years (range: 18–87), a median weight of 85 kg (range: 70–184 kg), and a median height of 1.78 m (range: 1.68–1.98 m) were included. Mean heart rate during MRI acquisition was 66 bpm (range: 39–99 bpm). Clinical indications for the cardiac MRI exams were known or suspected ischemic heart disease (*n* = 6), cardiomyopathy (*n* = 6), and myocarditis (*n* = 8). Structural cardiac abnormalities detected in the patient cohort included, but were not limited to, pacemaker devices and left ventricular wall aneurysms. Examples are shown in Figure 1. Demographic characteristics of the study population are summarized in Table 1.

**TABLE 1:** Demographic parameters of the study population.

Demographic parameters	All participants	Men	Women
Healthy cohort			
Number	57	28	29
Age (years)	42 (23–71)	44 (23–71)	40 (23–64)
Weight (kg)	76 (50–100)	85 (65–100)	67 (50–95)
Height (m)	1.75 (1.59–1.96)	1.82 (1.71–1.96)	1.69 (1.59–1.81)
BMI (kg/m^2^)	24.8 (18.0–33.2)	25.1 (20.2–31.8)	23.5 (18.0–33.2)
Heart rate (bpm)	68 (44–93)	66 (44–86)	68 (49–93)
Patient cohort			
Number	20	15	5
Age (years)	63.5 (18–87)	64 (18–87)	57 (44–74)
Weight (kg)	85 (70–184)	85 (70–184)	77 (73–91)
Height (m)	1.78 (1.68–1.98)	1.8 (168–1.98)	1.75 (1.68–1.80)
BMI (kg/m^2^)	27 (24.8–46.9)	27.8 (22.8–46.9)	26.9 (23.8–31.9)
Heart rate (bpm)	66 (39–99)	66 (39–99)	71 (53–83)

*Note*: Demographic data of study population. Values are presented as median with minimum and maximum values in brackets.

Abbreviation: BMI, body mass index.

### 3.2 Subjective Image Analysis

**FIGURE 2:**
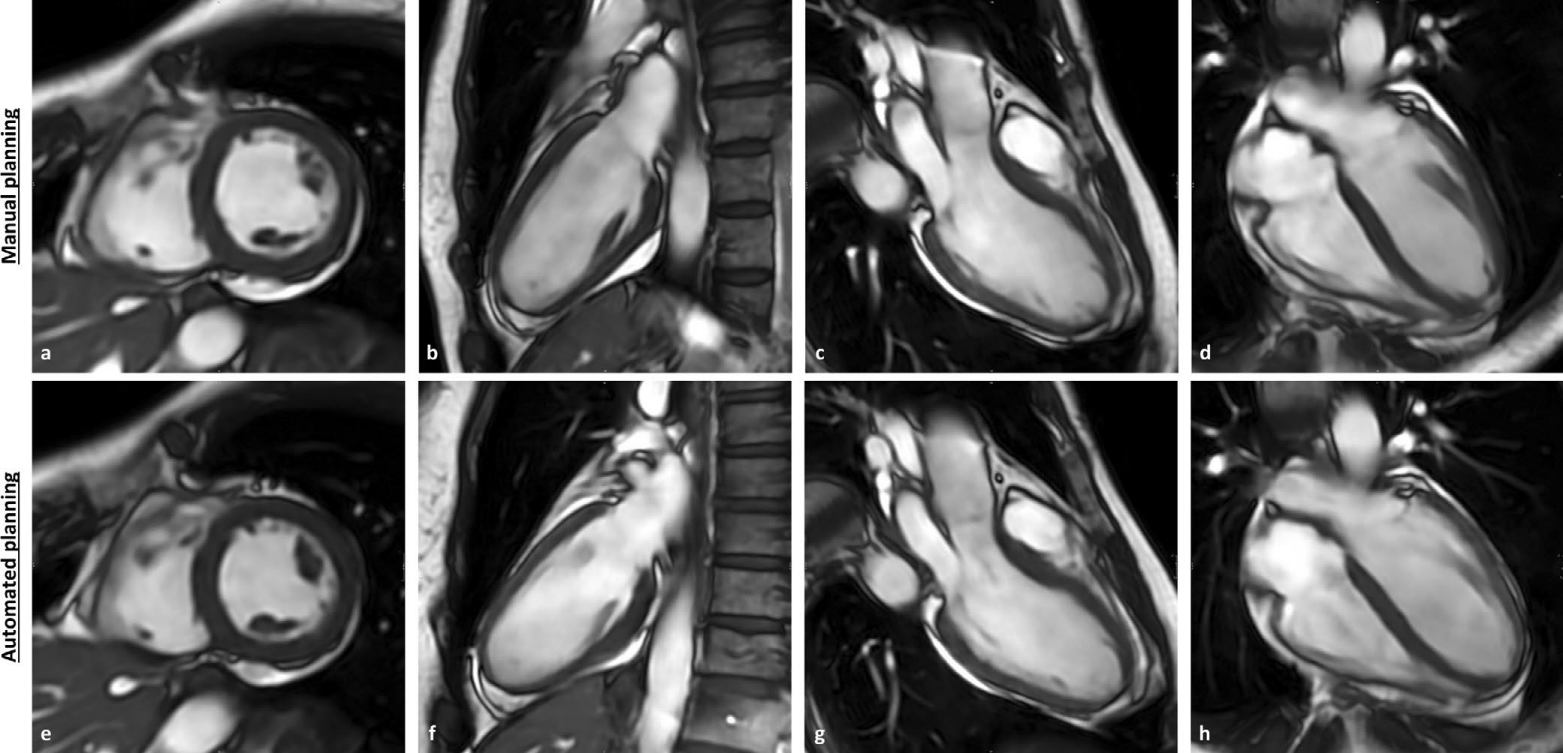
Cine‐sequence image stacks from a 55 year old female. The upper row shows images with manual plane prescription, the bottom row shows images acquired using the fully automated planning approach. (a,e) Short‐axis images in mid‐ventricular position, (b,f) 2‐chamber‐view images, (c,g) 3‐chamber‐view images, and (d,h) 4‐chamber‐view images.

The overall image quality was rated good to excellent for all manually and automatically planned image series by both readers with a mean average rating of 4.77/4.80 (*p* = 0.231) for contrast, 4.21/4.18 (*p* = 0.492) for blurring and 4.71/4.73 (*p* = 0.312) for artifacts. Figure 2 shows an example from manual and automated planned imaging stacks in all orientations.

The image plane positioning quality ratings of the SAX image stacks, 2CH, 3CH, and 4CH views are presented in Table 2. The highest mean average quality rating was observed for the SAX and 4CH image series for both manual and automated prescription. There were no significant differences in image quality scores between automatic and manual planning for any image pane orientation (SAX: *p* = 0.845; 2CH: *p* = 0.634; 3CH: *p* = 0.343; 4CH: *p* = 0.783).

**TABLE 2:** Subjective image quality scores from manually and automatically planned planes.

	Manual planning	Automated planning	*p*
Subjective image quality			
SAX	4.81	4.79	0.845
2CH	4.43	4.39	0.634
3CH	4.54	4.64	0.343
4CH	4.70	4.75	0.783
Overall	4.62	4.64	0.812

*Note*: Mean subjective image quality ratings of both readers (scale from 1 “*not diagnostic*” to 5 “*excellent*” image quality).

Abbreviations: 2CH, 2‐chamber‐view; 3CH, 3‐chamber‐view; 4CH, 4‐chamber‐view; SAX, short‐axis.

The results of the reacquisition analysis are summarized in Table S1. The number of cases with a need for reacquisition was lower for automatically prescribed image planes across all cardiac views compared to manually prescribed image planes. Overall, only 2.19% (5/228) of automatically prescribed image planes received a nondiagnostic or poor image plane positioning quality. In contrast, 5.26% (12/228) of manually prescribed image planes would require a reacquisition due to restricted quality of image plane positioning.

### 3.3 Plane Orientation Differences

**FIGURE 3:**
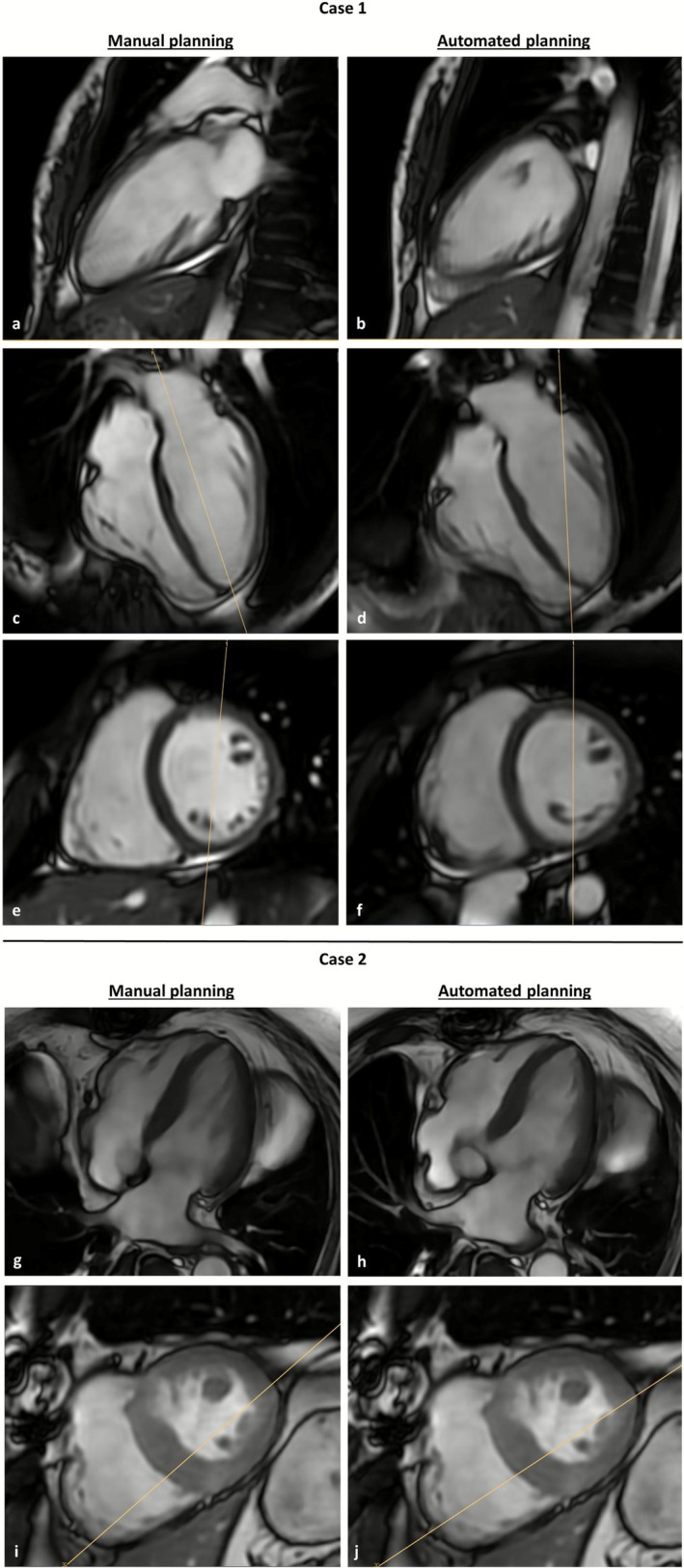
Cine‐sequence images from a 24 year old healthy female (a–f, case 1) and a 67 year old male patient with suspected ischemic heart disease (g–j, case 2). Case 1: (a) Manually prescribed 2CH view with a 5/5 quality rating. (c,e) Corresponding manually prescribed 4CH view and SAX view with the position of the 2CH view from (a) superimposed. (b) Automatically prescribed 2CH view with a 2,5/5 quality rating. (d,f) Corresponding automatically prescribed 4CH view and SAX view with the position of the 2CH view from (b) superimposed. In this case the automated planning tool detected the RV apex instead of the LV apex. The angle deviation between manually and automatically prescribed 2CH image planes was 22°. Case 2: The upper row illustrates the 4CH image plane which was initially acquired with automated plane positioning (h) and subsequently with manual correction (g). The bottom row shows the 4CH image plane on SAX images, highlighting the suboptimal angulation of the automated prescribed plane (j). The manually corrected image plane cuts through the greatest extent of the RV as recommended by established imaging guidelines (i). 2CH, 2‐chamber‐view; 4CH, 4‐chamber‐view; RV, right ventricle; SAX, short‐axis; LV, left ventricle.

The absolute differences between manual and automated prescribed image planes are shown in Table 3, together with equivalent data obtained in previously published studies. The lowest mean angle deviation between plane orientations was observed for SAX image stacks and the 4CH view images. The highest mean angle deviation was observed for the 2CH view planes (10.3° ± 5.8°). In two cases, the 2CH mean angle difference exceeded 20°. In one of these (Figure 3), the manual prescribed images received an average rating of 5/5, while the automated prescription received an average rating of 2.5/5. In the second case, the manual prescription received a rating of 3.5/5, while the automated prescription received a rating of 5/5.

**TABLE 3:** Mean angulation discrepancies between manual and automated prescription.

	SAX	2CH	3CH	4CH
AIRxHeart prototype	6.7 ± 4.3	10.3 ± 5.8	8.9 ± 5.1	8.0 ± 4.8
Wei et al. [17]	5.5 ± 3.5	4.8 ± 3.1	6.2 ± 3.9	6.3 ± 4.0
Edalati et al. [16]	6.2 ± 3.3	5.8 ± 2.5	5.5 ± 3.5	4.4 ± 1.4
Blansit et al. [15]	4.9 ± 4.9	6.5 ± 6.3	9.0 ± 8.8	5.2 ± 3.8
Nitta et al. [14]	3.1 ± 1.7	7.3 ± 4.8	5.8 ± 3.8	4.5 ± 3.8
Frick et al. [12]	6.7 ± 3.6	7.1 ± 3.6	9.1 ± 6.3	7.7 ± 6.1
Lu et al. [13]	8.6 ± 9.7	18.9 ± 21.0	12.3 ± 11.0	17.6 ± 19.2

*Note*: Mean angle deviation of imaging planes between automated and manual prescribed planes. Results are also presented from previously published studies for comparison. Numbers represent absolute mean degrees ± standard deviation.

Abbreviations: 2CH, 2‐chamber‐view; 3CH, 3‐chamber‐view; 4CH, 4‐chamber‐view; SAX, short‐axis.

### 3.4 Volumetric Analysis

**FIGURE 4:**
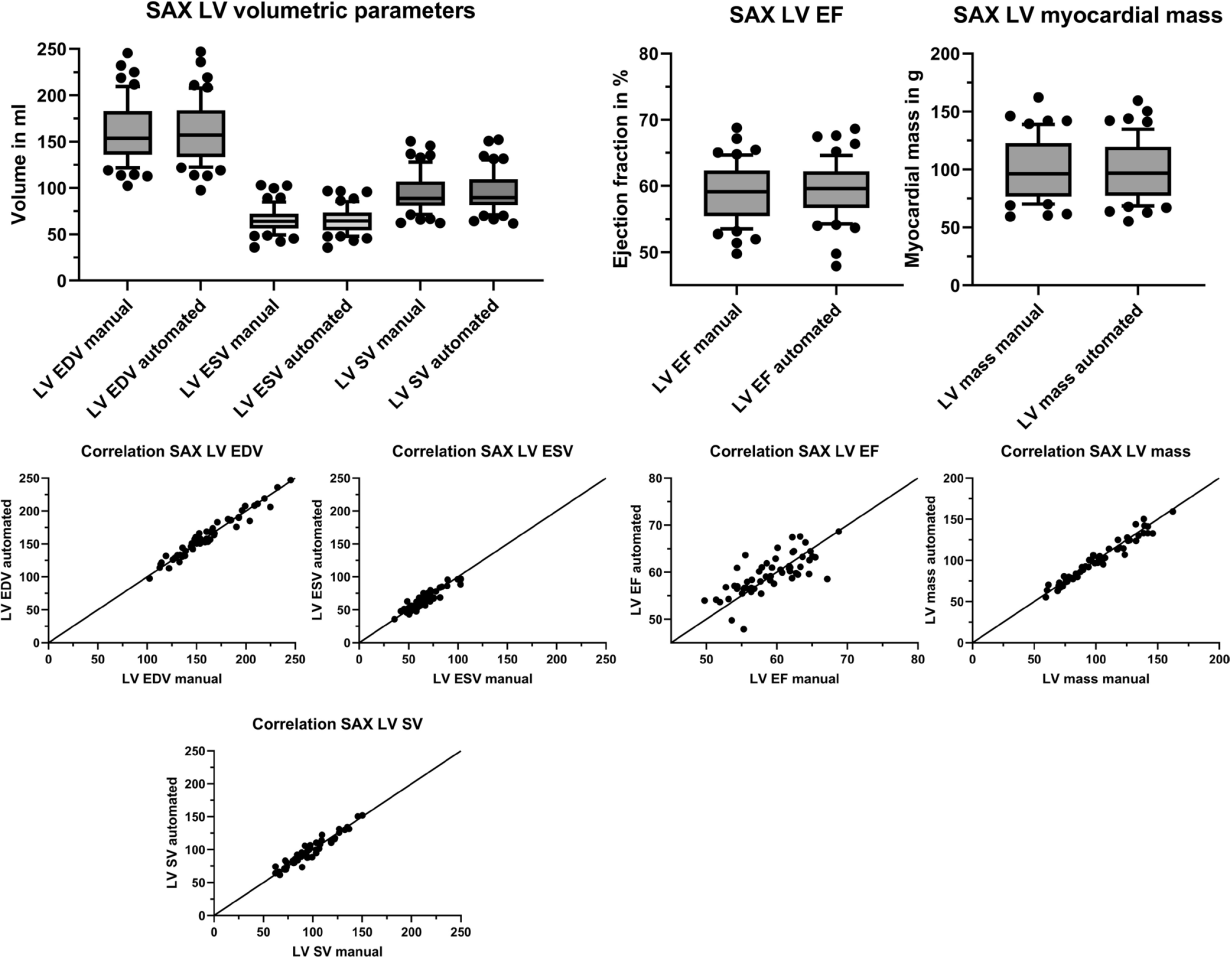
Left ventricular volumetric parameters derived from short axis analysis. Upper row: volumetric parameters derived from short axis image stacks including end‐diastolic volume, end‐systolic volume, stroke volume, ejection fraction, and myocardial mass. Bottom row: correlation plots derived from nonparametric Spearman correlation analysis. EDV, end‐diastolic volume; ESV, end‐systolic volume; EF, ejection fraction; LV, left ventricle; SAX, short‐axis; SV, stroke volume.

The volumetric parameters from short‐axis and biplane assessments of manually and automatically planned images are summarized in Table 4. All volumetric values of the LV and RV were within the normal reference ranges [8]. There were no significant differences in the short axis derived LV EDV (manual: 153.7 mL; automated: 157.0 mL; *p* = 0.305) and LV ESV (manual: 64.1 mL; automated: 64.5 mL; *p* = 0.683). Similarly, no significant deviations were observed for the biplane derived LV EDV (manual: 151.6 mL; automated: 148.3 mL; *p* = 0.745) and LV ESV (manual: 56.4 mL; automated: 57.1 mL; *p* = 0.712). There were also no significant differences in short axis derived RV EDV (manual: 170.6 mL; automated: 164.6 mL; *p* = 0.301) and RV ESV (manual: 74.6 mL; automated: 77.0 mL; *p* = 0.787). The parameters LV SV (SAX‐derived: *p* = 0.168; biplane‐derived: *p* = 0.795), LV EF (SAX‐derived: *p* = 0.215; biplane‐derived: *p* = 0.686), RV SV (SAX‐derived: *p* = 0.601), and RV EF (SAX‐derived: *p* = 0.695), which were derived from EDV and ESV, respectively, as well as LV mass (SAX‐derived: *p* = 0.173; biplane‐derived: *p* = 0.145), also showed no significant differences between the planning modes. Correlation analysis revealed a very high positive correlation with Spearman's *ρ* > 0.9 for all LV and RV volumetric parameters derived from SAX and biplane images except for LV EF and RV EF which showed high positive correlation with Spearman's *ρ* > 0.75. The distribution of LV volumetric parameters for both manual and automated planning as well as corresponding correlation plots are shown for SAX derived parameters in Figure 4 and for biplane derived parameters in Figure S1.

**TABLE 4:** Short‐axis and biplane volumetric results.

	Manual planning			Automated planning			Automated vs. manual planning	
	Median	Range (min/max)		Median	Range (min/max)		*p*	Spearman's *ρ*
Short‐axis volumetric results								
Left ventricle (LV)								
LV EDV (mL)	153.7	102.4	245.5	157.0	97.5	247.0	0.305	*0.973*
LV ESV (mL)	64.1	35.8	103.0	64.5	35.8	96.7	0.683	*0.898*
LV SV (mL)	88.8	62.2	150.3	89.6	61.8	152.0	0.168	*0.945*
LV EF (%)	59.1	49.8	68.8	59.6	47.9	68.6	0.215	*0.763*
LV mass (g)	96.3	59.4	162.2	96.8	55.2	159.4	0.173	*0.976*
Right ventricle (RV)								
RV EDV (mL)	170.6	96.7	289.3	164.6	98.7	299.2	0.301	*0.949*
RV ESV (mL)	74.6	36.5	142.0	77.0	35.5	147.6	0.787	*0.909*
RV SV (mL)	88.3	57.4	147.8	90.0	56.8	151.7	0.601	*0.910*
RV EF (%)	55.3	42.1	66.2	55.1	42.7	71.3	0.695	*0.751*
Biplane volumetric results								
Left ventricle (LV)								
LV EDV (mL)	151.6	97.8	238.2	148.3	98.7	249.2	0.745	*0.978*
LV ESV (mL)	56.4	37.4	92.3	57.1	37.2	93.0	0.712	*0.952*
LV SV (mL)	94.7	55.1	154.2	91.8	58.2	156.2	0.795	*0.950*
LV EF (%)	62.7	54.3	75.1	63.3	53.8	69.9	0.686	*0.797*
LV mass (g)	111.2	72.0	178.1	109.2	72.0	178.7	0.145	*0.981*

*Note*: Volumetric parameters from short‐axis and biplane analyses. Values are medians with corresponding minimums and maximums. *p*‐values were calculated using nonparametric Wilcoxon matched pairs signed rank test and correlation was analyzed using the nonparametric Spearman correlation analysis (for all Spearman's ρ printed in italics *p < 0.001)*.

Abbreviations: EDV, end‐diastolic volume; EF, ejection fraction; ESV, end‐systolic volume; LV, left ventricle; RV, right ventricle; SV, stroke volume.

**FIGURE 5:**
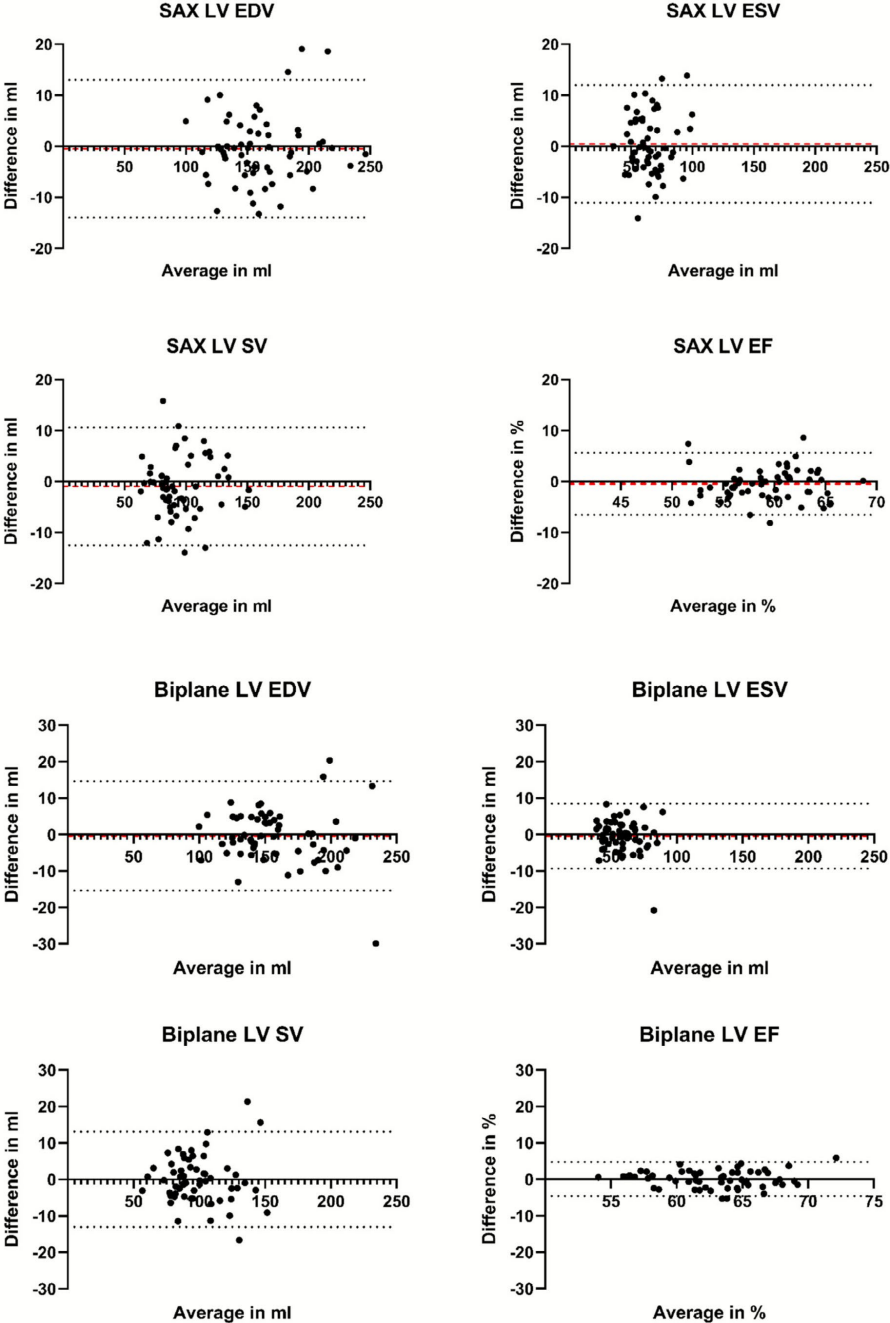
Bland–Altman plots for left ventricular volumetric short‐axis and biplane parameters. The red dashed lines represent the mean bias, the black dotted lines the 95% limits of agreement. EDV, end‐diastolic volume; EF, ejection fraction; ESV, end‐systolic volume; LV, left ventricle; SAX, short‐axis; SV, stroke volume.

Bland–Altman analysis showed minor discrepancies between the volumetric parameters derived from the automated planned imaging stacks and those obtained through manual plane prescription. Bland–Altman plots of LV EDV, ESV, SV, and EF for volumetric analysis of short‐axis image stacks and for biplane assessment are shown in Figure 5. The Bland–Altman plots for the RV volumetric parameters are shown in Figure S2. Short axis LV EDV showed a mean bias of −0.5 mL (95% limits of agreement (LoA): −14.0 mL/13.0 mL) and LV ESV showed a mean bias of 0.5 mL (95% LoA: −11.0 mL/12.0 mL), whereas short axis RV EDV showed a mean bias of −0.9 mL (95% LoA: −24.8.0 mL/23.1 mL) and RV ESV, a mean bias of −0.1 mL (95% LoA: −18.2 mL/18.2 mL). The results of the Bland–Altman analysis for short axis and biplane volumetric parameters are presented in Table 5.

**TABLE 5:** Bland–Altman analysis of short‐axis and biplane volumetric parameters.

	Manual vs. automated plane prescription		
	Bias	SD of bias	95% limits of agreement
Bland–Altman analysis of short axis volumetric parameters			
Left ventricle (LV)			
LV EDV (mL)	−0.5	6.9	−14.0/13.0
LV ESV (mL)	0.5	5.9	−11.0/12.0
LV SV (mL)	−1.0	5.9	−12.5/10.6
LV EF (%)	−0.4	3.1	−6.5/5.6
Right ventricle (RV)			
RV EDV (mL)	−0.9	12.2	−24.8/23.1
RV ESV (mL)	−0.1	9.3	−18.2/18.2
RV SV (mL)	−0.8	7.3	−15.1/13.5
RV EF (%)	−0.2	3.5	−7.0/6.7
Bland–Altman analysis of biplane volumetric parameters			
Left ventricle (LV)			
LV EDV (mL)	−0.4	7.7	−15.4/14.6
LV ESV (mL)	−0.4	4.5	−9.3/8.5
LV SV (mL)	0.1	6.7	−13.0/13.1
LV EF (%)	0.1	2.4	−4.6/4.8

*Note*: Bland–Altman analysis of short‐axis and biplane volumetric analysis comparing the automatically planned images with the manually planned images (reference standard).

Abbreviations: EDV, end‐diastolic volume; EF, ejection fraction; ESV, end‐systolic volume; LV, left ventricle; RV, right ventricle; SD, standard deviation; SV, stroke volume.

### 3.5 Application to a Clinical Patient Cohort

Automated plane prescription was conducted for four cardiac views (SAX, 2CH, 3CH, and 4CH) per patient (*n* = 20), resulting in a total of 80 image planes. Volumetric assessment of the LV resulted in the following mean LV parameters: LV EDV 175.9 mL (range: 100–419 mL), LV ESV 81.5 mL (range: 18–312 mL), LV SV 94.4 mL (range: 55–191 mL), LV EF 57.6% (range: 26%–82%), and LV mass 139.6 g (range: 77–210 g).

The automated plane prescription tool demonstrated a high efficacy, with no recorded total planning errors. In 91.2% of clinical scans (73/80) no manual corrections were necessary, whereas in 8.8% of cases (7/80) manual adjustments were required. In four cases (4/80, 5%) the automatically determined number of slices in the SAX was one or two too low for full coverage of the LV in diastole. In the remaining three cases (3/80, 3.8%) minor manual adjustments to the plane's angle were necessary, as shown in Figure 3. All adjustments were made in 4CH view planes.

## 4 Discussion

This prospective cohort study evaluated a fully automated AI‐based plane positioning tool for cardiac MRI, comparing it to manual plane prescription as the reference standard. The automatically prescribed imaging planes (SAX, 2CH, 3CH, and 4CH views) provided datasets with good to excellent subjective image quality without significant differences compared to manual planning. Mean average angulation differences were < 10° with the largest deviation occurring for the 2CH view. The volumetric assessment utilizing short‐axis and biplane analyses showed no significant differences between automated and manual plane positioning. Additionally, the automated plane prescription demonstrated a robust performance in a clinical pilot study, with no need for manual corrections in over 90% of cases.

The fully automated planning approach in our study resulted in good to excellent image quality which was not significantly different from the image quality achieved by manual planning. This is in line with the findings reported by Frick et al. [12] and those of a recently published study in a clinical patient cohort by Glessgen et al. [19]. Similar to the observations of Frick and colleagues [12], the best rating was recorded for short‐axis image stacks.

The highest difference between manual and automatic planned images was observed for the 2CH view. A subsequent review of the two cases with the highest angle deviations in the 2CH view revealed larger differences in the subjective ratings of the image plane positioning quality. In one case, the image plane quality was better for the manually planned images whereas in the second case the quality was better for the automatically planned image plane. Lower mean angulation values were found for the 3CH view and 4CH view. Recent studies by Blansit et al. [15] and Wei et al. [17] have reported lower absolute mean angulation differences for all imaging planes compared to our findings. However, both of these studies were conducted in a retrospective manner and automatic plane prescription was computed without subsequently acquiring cine data. The effect of automatic plane prescription on image quality could therefore not be assessed. Mean angulation differences reported by prospective studies from Frick et al. [12] and Edalati et al. [16] show results similar to our study, while an automatic plane positioning model proposed by Nitta et al. [14] reached lower mean angulation discrepancies over all image planes in a small prospective cohort. While the manual planning approach is regarded as the reference standard, it should be noted that it is influenced by the experience of the technicians performing the scans. In the present study, a group of technicians with varying levels of experience were assigned to perform manual planning during the MRI acquisitions, reflecting the conditions in daily clinical care. In contrast, in the study of Frick et al. [12], all MRI exams were planned by a technologist with over 8 years of experience who performed more than 10 cardiac exams per day. Consequently, the comparability of the absolute mean angulation discrepancies between studies is limited. However, the low absolute mean angle differences and high subjective image quality ratings achieved in this study indicate that the fully automated plane prescription tool appears to provide high‐quality imaging planes.

The functional assessment of the ventricles provided by volumetric analysis is important for the diagnostic value and quality of a cardiac MRI exam. In the present study, a volumetric assessment using both short‐axis and biplane analyses showed a high degree of consistency across all volumetric parameters, with no significant differences observed between automated and manual plane positioning techniques. The Bland–Altman analysis revealed nonsignificant mean bias and small LoAs for LV EDV or LV ESV in both SAX and biplane volumetric assessments. For SAX volumetric results, the majority of these outliers were likely attributable to variations in basal slice inclusion, whereas outliers in the biplane analysis were most likely caused by differing angles between manually and automatically prescribed planes. However, these findings underline the suitability of the evaluated plane positioning tool for clinical application maintaining the high diagnostic quality required for cardiac MRI. A prospective study by Frick et al. [12] demonstrated the feasibility of an automated planning approach several years ago. However, the software utilized required the acquisition of an additional survey scan to execute the automated plane prescription. Furthermore, the software initially failed to perform the planning in nearly one‐third of scans, necessitating the acquisition of an additional survey scan. In contrast, the plane positioning tool proposed in the present study does not require the acquisition of additional survey scans and uses the most recently acquired cine images. This could be useful in cases after minor patient movements, when the initial survey scans no longer provide accurate geographical information and must be reacquired. The proposed planning tool promises to be more robust against small patient movements. Additionally, the tool demonstrated overall a very high success rate in volunteer and clinical MRI exams. Reliable performance of an automated system is fundamental for clinical implementation, in order to avoid the need for scan repetitions and troubleshooting. The automated plane prescription tool was also successfully tested in a subsequent pilot study including 20 patients in a clinical setting. A high technical reliability was observed and in over 90% of cases, manual corrections were not required. In the remaining scans, only minor manual adjustments were necessary. The purpose of the automated planning tool is to provide assistance to the technicians, especially those with limited experience in cardiac MRI, which may increase the scan efficacy and availability. It is not intended to fully automate the entire cardiac MRI scan.

A recently published randomized prospective study by Glessgen et al. [19] underlined the potential of automated planning with a focus on efficacy measurements. The study reported a substantial reduction in idle time, defined as the time required for plane positioning and adaptations of scanner settings, and an increase in error‐free examinations compared to manual planning performed by technicians with varying levels of experience (from low experience [< 2 years] to high experience [> 5 years]). In contrast, the present study provides an intraindividual comparison focusing on the plane prescription procedure itself and its impact on the volumetric analysis. The standard cardiac plane positions generated automatically or manually for the SSFP cine sequences are typically propagated to other subsequently acquired cardiac MRI sequences, such as late‐gadolinium enhancement or mapping sequences. Consequently, the automated planning may also beneficially impact the prescription of these sequences.

The findings of Glessgen et al. [19] and our study have implications for the application of automated planning for cardiac MRI. Despite the differences in methodology, focus, and utilization of different automated planning tools developed by different vendors, both investigations show the potential of automated planning to improve efficacy in the clinical setting and demonstrate the potential to accelerate cardiac MRI acquisition while maintaining diagnostic quality and reducing error rates.

## 5 Limitations

Firstly, the possibility of observer bias influencing the manual planning procedure should be considered, given that the technologists were not blinded to the automated planning process and did not change between both planning approaches. The automated planning was performed first to ensure an error‐free start of the evaluated early version of the prototype software. Secondly, the study was conducted on a single 1.5 T MRI scanner system during all cardiac MRI exams. This limits the generalizability of the findings to other scanner systems from different vendors and to 3 T field strength. Finally, the automated planning tool was only tested in a small number of patients (*n* = 20) under clinical conditions and with a limited number of cardiac structural abnormalities including pacemaker devices. Although the results in this sub‐cohort were promising, the performance and robustness of the automated planning need to be evaluated in more complex nonphysiological anatomical conditions of the heart, such as patients with congenital heart diseases before and after surgical treatment.

## 6 Conclusion

This study demonstrated that fully automated plane positioning for cardiac MRI may provide high‐quality images, with derived ventricular volumetric parameters being not significantly different from those achieved following manual planning. This has the potential to simplify the acquisition process and improve the availability of cardiac MRI in sites with less specialized technologists.

## Supporting information


**Figure S1:** Left ventricular volumetric parameters derived from biplane analysis. Upper row: volumetric parameters derived from 2CH and 4CH view long axis images including end‐diastolic volume, end‐systolic volume, stroke volume, ejection fraction and myocardial mass. Bottom row: correlation plots derived from nonparametric spearman correlation analysis. 2CH, 2‐chamber; 4CH, 4‐chamber; EDV, end‐diastolic volume; EF, ejection fraction; ESV, end‐systolic volume; LV, left ventricle; SAX, short‐axis; SV, stroke volume.

**Figure S2:** Bland–Altman plots for right ventricular volumetric short‐axis volumetric parameters. The red dashed line represents the mean bias, the black dotted lines the 95% limits of agreement. EDV, end‐diastolic volume; EF, ejection fraction; ESV, end‐systolic volume; RV, right ventricle; SV, stroke volume.

**Table S1:** Reacquisition analysis of manually and automatically prescribed image planes.
