# Supplementary figures and images for: Fully Automated Plane Prescription in Cardiac MRI: A Prospective Cohort Study
Source: J Magn Reson Imaging. 2025 Nov 30;63(3):891–903. doi: 10.1002/jmri.70178 (PMC12891750; doi:10.1002/jmri.70178)

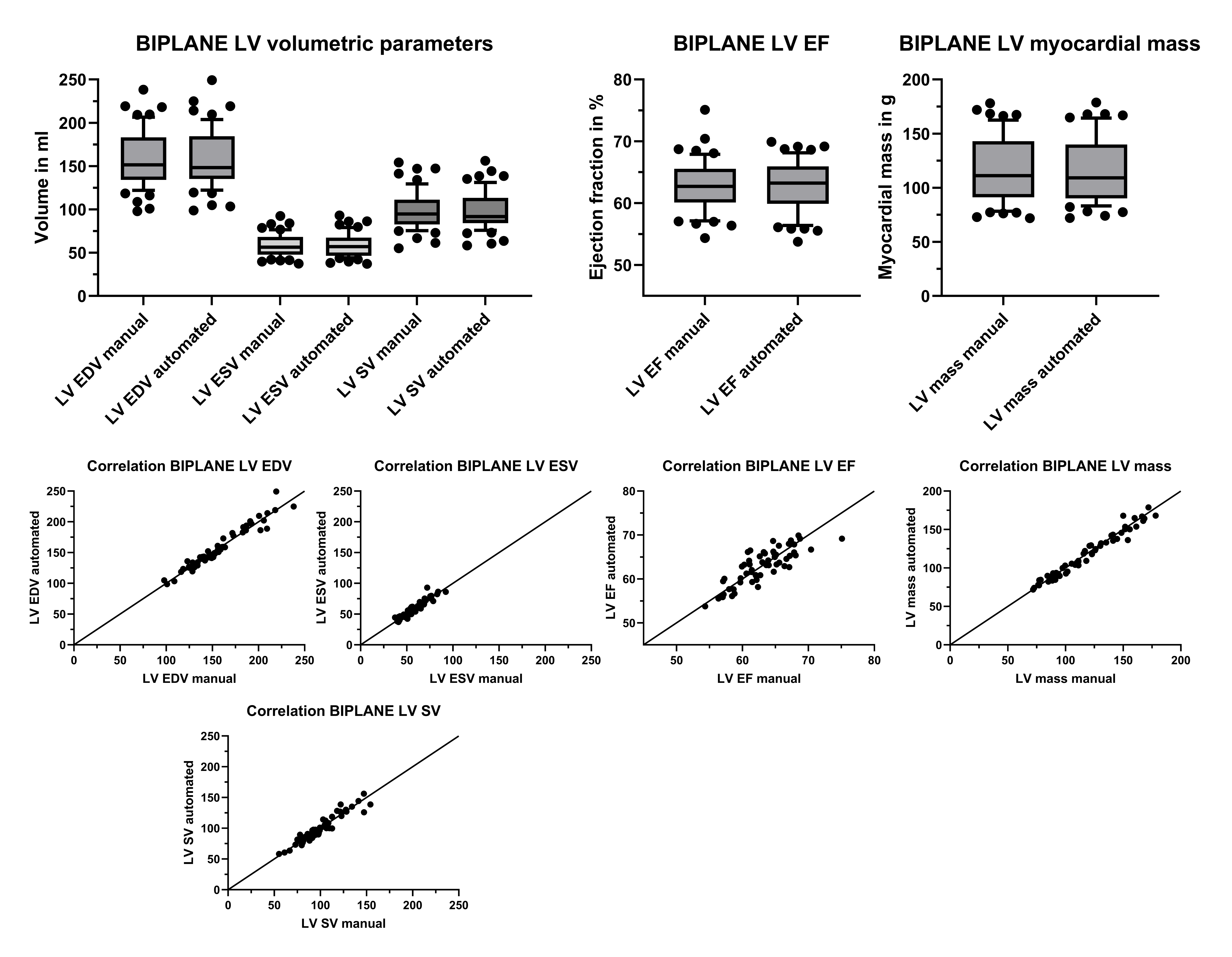

Supplement: Supplementary file 1 — Figure S1: Left ventricular volumetric parameters derived from biplane analysis. Upper row: volumetric parameters derived from 2CH and 4CH view long axis images including end‐diastolic volume, end‐systolic volume, stroke volume, ejection fraction and myocardial mass. Bottom row: correlation plots derived from nonparametric spearman correlation analysis. 2CH, 2‐chamber; 4CH, 4‐chamber; EDV, end‐diastolic volume; EF, ejection fraction; ESV, end‐systolic volume; LV, left ventricle; SAX, short‐axis; SV, stroke volume. [file JMRI-63-891-s001.tif]

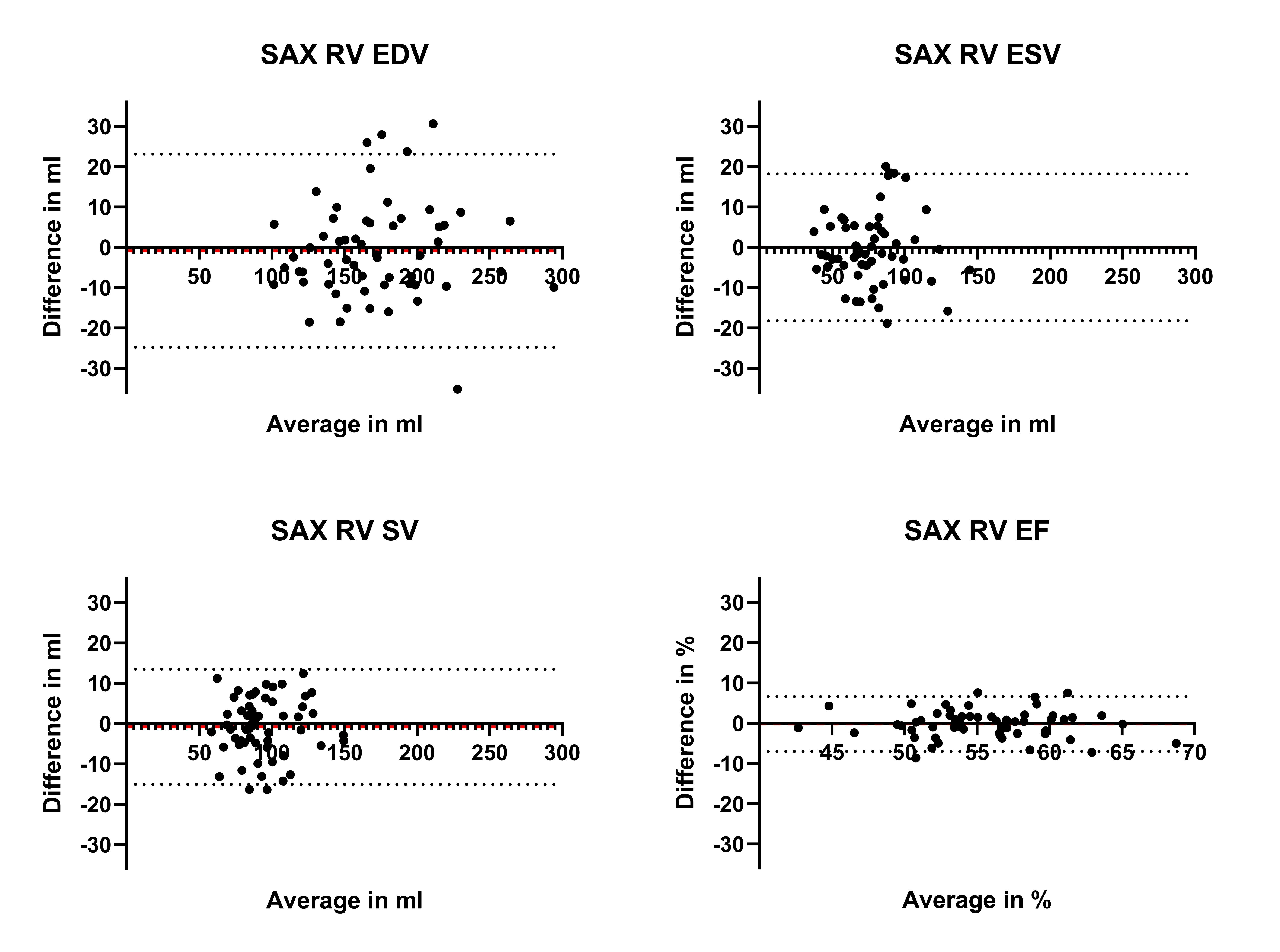

Supplement: Supplementary file 2 — Figure S2: Bland–Altman plots for right ventricular volumetric short‐axis volumetric parameters. The red dashed line represents the mean bias, the black dotted lines the 95% limits of agreement. EDV, end‐diastolic volume; EF, ejection fraction; ESV, end‐systolic volume; RV, right ventricle; SV, stroke volume. [file JMRI-63-891-s003.tif]
