# Supplementary material for: Fully Automated Plane Prescription in Cardiac MRI: A Prospective Cohort Study
Source: J Magn Reson Imaging. 2025 Nov 30;63(3):891–903. doi: 10.1002/jmri.70178 (PMC12891750; doi:10.1002/jmri.70178)
Supplement: Supplementary file 3 — Table S1: Reacquisition analysis of manually and automatically prescribed image planes. [file JMRI-63-891-s002.docx]

## **Supplemental Table 1: Reacquisition analysis of manually and automatically prescribed image planes**

|  | **Manual planning** | | **Automated planning** | |
| --- | --- | --- | --- | --- |
|  | Total number | percentage | Total number | percentage |
| SAX (n = 57) | 0 | 0% | 0 | 0% |
| 2CH (n = 57) | 2 | 3.51% | 1 | 1.75% |
| 3CH (n = 57) | 6 | 10.53% | 2 | 3.51% |
| 4CH (n = 57) | 4 | 7.02% | 2 | 3.51% |
| Overall (n = 228) | 12 | 5.26% | 5 | 2.19% |

***Supplemental Table 1: Reacquisition analysis of manually and automatically prescribed image planes. Number of cases which received a “non-diagnostic” (1/5) or “poor” (2/5) subjective quality rating of the plane positioning was documented for each cardiac image plane and overall image planes. These number of cardiac planes are considered insufficient and would require correction of plane positioning and a reacquisition in a clinical setting. SAX – short-axis; 2CH – 2-chamber-view; 3CH – 3-chamber-view; 4CH – 4-chamber-view.***
